# Household Transmission of COVID-19 and Influenza During the Early Omicron Era: An Insurance Claims Database Study in Japan

**DOI:** 10.3390/v18030324

**Published:** 2026-03-05

**Authors:** Kosaku Komiya, Shogo Miyazawa, Yuki Yoshida, Satoshi Kojima, Akihiko Hagiwara, Yoshitake Kitanishi

**Affiliations:** 1 Respiratory Medicine and Infectious Diseases, Faculty of Medicine, Oita University, Oita 879-5593, Japan; 2 Data Science Department, Shionogi & Co., Ltd., Osaka 530-0011, Japan; yuuki.yoshida@shionogi.co.jp (Y.Y.); yoshitake.kitanishi@shionogi.co.jp (Y.K.); 3 Medical Affairs Department, Shionogi & Co., Ltd., Osaka 530-0011, Japan; satoshi.kojima@shionogi.co.jp

**Keywords:** household transmission, health insurance claims database, infection control, epidemiology

## Abstract

Recurrent outbreaks of coronavirus disease 2019 (COVID-19) in Japan highlight the continued need for effective prevention, particularly household transmission. This study clarifies the epidemiological profile of household transmission of COVID-19 and influenza by quantifying transmission rates according to demographic and relational characteristics. An anonymized health insurance claims database was used to identify index patients diagnosed with COVID-19 or influenza between November 2021 and August 2023. Transmission events were defined as cases in which household members were diagnosed with the same infection in the week following the index patient’s diagnosis (Day 1). Analyses were stratified by the age of the index patients and age-based transmission patterns between index patients and household members. A total of 1,001,509 index patients with COVID-19 and 207,090 with influenza met the inclusion criteria. Transmission rates were highest among index patients aged <12 years (COVID-19, 40.17%; influenza, 33.51%). Transmission from children was disproportionately high, while age-combination analysis demonstrated elevated transmission both from and to children for both diseases. COVID-19 exhibited increased transmission among adults. As the primary drivers of household transmission for both COVID-19 and influenza, preventive strategies should be tailored to children. COVID-19 interventions must also address transmission among adults to strengthen household-level infection control.

## 1. Introduction

Since its emergence in late 2019, coronavirus disease 2019 (COVID-19), caused by severe acute respiratory syndrome coronavirus 2 (SARS-CoV-2), has remained a persistent global health challenge. In Japan, widespread vaccination and the predominance of less virulent variants have led to declining public vigilance, accompanied by relaxation of basic non-pharmaceutical interventions such as mask-wearing and hand hygiene. Public vigilance has declined, but COVID-19 continues to generate recurrent epidemic waves. In 2024, the 11th and 12th epidemic waves produced substantial case numbers and severe outcomes, particularly among older adults, underscoring the ongoing importance of monitoring transmission dynamics [1].

Household transmission has been recognized as a major pathway of SARS-CoV-2 spread and remains a critical focus for infection control, even during periods of restricted community interaction. The World Health Organization emphasizes that transmission occurs predominantly through close contact with respiratory droplets and aerosols, particularly in poorly ventilated indoor environments [2]. Modeling studies have demonstrated that post-return home behaviors and intrafamilial contact patterns strongly influence secondary transmission within households [3]. Empirical evidence further indicates that household transmission is shaped by the age of the index case. For instance, Canadian studies have reported higher transmission from younger children [4], while multinational investigations across pediatric hospitals in Canada and the United States revealed that asymptomatic children, especially those under the age of 5, contributed disproportionately to household spread, resulting in elevated secondary attack rates [5]. However, most of these investigations were conducted during the Delta and early Omicron periods, and large-scale studies capturing household transmission during subsequent, more extensive Omicron waves, remain limited. Identifying the demographic and relational factors that drive household spread in the Omicron period is therefore essential to inform targeted preventive strategies.

Influenza, another viral respiratory infection of major global significance, provides a valuable comparator. During the 2009 A(H1N1) pandemic, children aged 5 to 17 years demonstrated the highest household transmission rates [6,7], and school-age children have consistently been identified as central to propagating influenza within households [8]. Compared with COVID-19, the household transmission dynamics of influenza have been more comprehensively characterized, with evidence consistently highlighting children as the principal introducers and amplifiers of infection in domestic settings.

COVID-19 and influenza are respiratory viruses that frequently circulate during overlapping seasons, share similar clinical manifestations, and exhibit comparably high household transmission rates [9]. Preventive measures within the household, including isolation, ventilation, and hygiene practices, are largely common to both infections. Nevertheless, no study has comprehensively evaluated household transmission of these two viral pathogens during the same observation period, particularly in the era dominated by Omicron variants.

Both diseases continue to exert significant public health burdens in Japan, where household transmission constitutes a major driver of sustained outbreaks. Characterizing the demographics associated with intrafamilial spread is therefore critical for developing tailored preventive strategies. This study was designed to address this gap by quantifying household transmission rates of COVID-19 and influenza using a large-scale health insurance claims database, with analyses stratified by the age of index patients and their household members. The resulting evidence is intended to inform the refinement of household-level infection control measures and to contribute to more effective strategies for mitigating the spread of both respiratory viruses.

## 2. Materials and Methods

### 2.1. Data Sources

Anonymized data were obtained from the JMDC Claims Database (JMDC Inc., Tokyo, Japan), which compiles a comprehensive and longitudinal dataset of medical claims for approximately 17 million insured individuals. The database includes inpatient and outpatient Diagnosis Procedure Combination records as well as prescription-dispensing claims. Each individual can be tracked across multiple medical institutions, including clinics and hospitals, provided continuous insurance enrollment is maintained.

In the Japanese health insurance system, each insured subscriber and their registered dependents under the same insurance contract share a unique insurance family code. This family code is consistently recorded in the claims database and enables linkage of individuals belonging to the same insurance unit. In this study, individuals sharing the same insurance family code were classified as members of the same household. This approach is widely used in Japanese administrative and claims-based research and is generally considered a reasonable proxy for household cohabitation.

### 2.2. Study Design and Population

This observational study using the Japanese claim database was conducted to estimate household transmission rates of COVID-19 and influenza. Eligible index patients were diagnosed with COVID-19 (ICD-10 codes U071, U072) or influenza (ICD-10 codes J09, J10, J11) between 30 November 2021 and 24 August 2023. In this claims-based study, the index patient was defined as the first individual diagnosed within a household during the study period. Day 1 was defined as the date of diagnosis, and follow-up was performed for the subsequent 8 days. The study period corresponded to the onset and continuation of the Omicron variant epidemic in Japan. In addition, much of this period coincided with comprehensive case reporting for COVID-19, during which testing was actively conducted [10]. Furthermore, influenza testing rates are generally high in Japan [11], indicating that this study period was a time when patient identification based on diagnosis was highly likely. Exclusion criteria were the absence of household members, hospitalization of the index patient on Day 1, occurrence of multiple index patients within the same household, and a diagnosis of the same infection (COVID-19 or influenza) within 180 days prior to Day 1 in either the index patient or household members. The first three criteria were applied to ensure that the index patient, defined as the first diagnosed individual within the household, is uniquely identifiable for the purpose of assessing subsequent household diagnoses. The fourth criterion was applied to exclude individuals with sufficient immunity due to a recent infection, which could alter susceptibility to reinfection and bias estimates of secondary transmission. Notably, for SARS-CoV-2, previous studies have demonstrated that natural immunity persists for at least approximately six months, supporting our choice of a 180-day window [12]. For individuals with multiple episodes of the same infection during the study period, only the first episode was analyzed.

This study was conducted in accordance with the Ethical Guidelines for Medical and Health Research Involving Human Subjects. Because only anonymized data were used, informed consent was not required (UMIN-CTR identifier: UMIN000059051).

### 2.3. Study Outcomes

The primary outcome was household transmission, defined as at least one household member receiving a diagnosis of the same infection between Day 2 and Day 8 after the index patient’s diagnosis. This transmission window was selected to cover the upper bound of the reported serial interval for COVID-19 and to allow for potential delays in secondary transmission [13,14]. In analyses stratified by age combinations, each household member was evaluated individually; multiple members within the same age category were treated as distinct observations.

### 2.4. Statistical Analyses

Household transmission rates were estimated separately for COVID-19 and influenza. Analyses were performed based on the age of index patients. Age-pair analyses of index patients and household members were conducted, standardized (mean = 0, variance = 1), and visualized using heatmaps.

Exploratory analyses assessed the interval from index diagnosis to secondary household infection, defined as the difference between the household member’s diagnosis date and Day 1. These analyses were stratified by index patient age and by index–household member age combinations.

All statistical analyses were performed using SAS Viya software (version 4.0 or higher; SAS Institute, Cary, NC, USA).

## 3. Results

### 3.1. Patients

**Figure 1:**
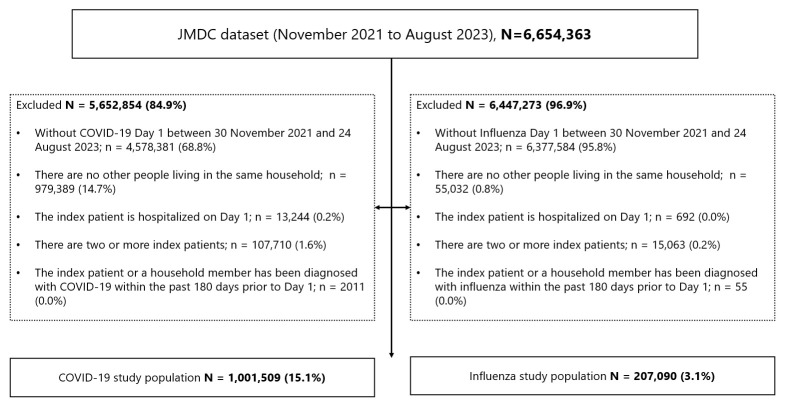
Flow diagram of the study population. Selection of index patients with COVID-19 and influenza from the JMDC claims dataset (November 2021–August 2023).

Between November 2021 and August 2023, a total of 1,001,509 index patients with COVID-19 and 207,090 index patients with influenza fulfilled the eligibility criteria (Figure 1). The baseline characteristics of the index patients on Day 1 are summarized in Table 1. In both cohorts, children younger than 12 years constituted the largest proportion of index patients. Marked differences between the two diseases were noted with respect to antiviral drug administration, which was substantially more common among influenza cases (Table 1).

### 3.2. Household Transmission Outcomes

Household transmission rates stratified by the age of index patients are presented in Table 2. Transmission was highest among index patients younger than 12 years, reaching 40.17% for COVID-19 and 33.51% for influenza.

**Figure 2:**
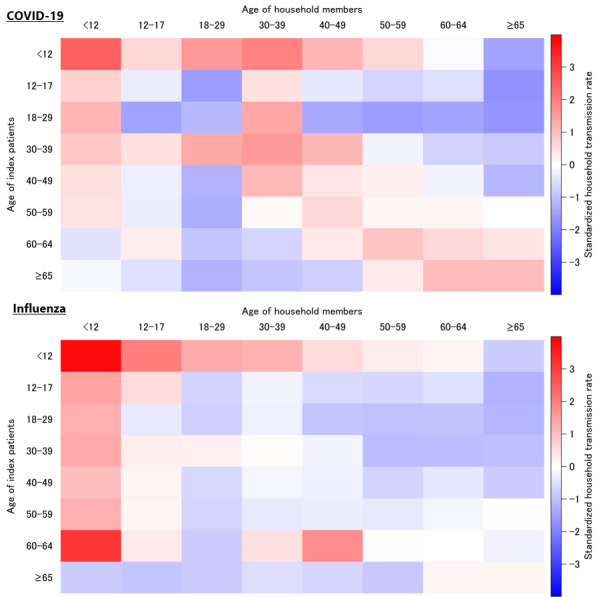
Standardized household transmission rates by age of index patients and household members. Heatmaps illustrating standardized household transmission rates for COVID-19 (**top**) and influenza (**bottom**) according to the age combinations of index patients and household members.

Age-combination analyses of index patients and household members are presented in Supplementary Table S1, with standardized transmission rates visualized in Figure 2. Both infections demonstrated a disproportionately high transmission from and to children. For COVID-19, elevated transmission was also evident between adults and among older adults, a pattern which was less pronounced for influenza.

### 3.3. Timing of Household Transmission

The mean interval from index diagnosis to secondary household infection is summarized in Table 3 and Supplementary Table S2. Across the entire study population, the average time to household transmission was 2.8 days for COVID-19 and 2.5 days for influenza. These intervals were consistent across most age groups of index patients, with only modest variation.

## 4. Discussion

This large-scale claims-based analysis from Japan provides comprehensive insights into the household transmission dynamics of COVID-19 and influenza. By examining transmission rates based on the age of index patients and their household members, the study clarifies the real-world patterns of intrafamilial spread during the Omicron-dominant period. Because this study relied on diagnosis dates from claims data, the index patient represents the first diagnosed individual rather than the true primary case. Therefore, observed age-specific transmission patterns should be interpreted as associations based on diagnostic sequence, not definitive evidence of transmission direction.

Transmission from children emerged as a consistent and prominent feature. Index patients younger than 12 years exhibited transmission rates exceeding 25% for both COVID-19 and influenza, the highest among all age groups. Analyses of age pairings further revealed that transmission both from and to children was disproportionately elevated, reinforcing the role of children as major drivers of intrafamilial spread. For COVID-19 specifically, additional high transmission rates were observed between adults and among older adults, patterns that were less evident for influenza. These results align with prior studies from Canada and the United States, which also identified children as key contributors to household spread, including during the Omicron epidemic period [4,5]. Notably, the present findings extend this evidence to the Japanese population, confirming that the predominance of child-to-child and child-to-adult transmission persisted even after the emergence of Omicron variants.

The age-related differences in household transmission can be partly explained by the challenges of applying preventive measures to children. Children often have a limited ability to recognize or report symptoms, and caregiving activities inherently involve close and prolonged contact, rendering isolation strategies impractical. In contrast, adults and older adults are generally more capable of implementing infection control behaviors, such as isolation and masking, once symptomatic. This likely contributed to the relatively lower influenza transmission rates observed in older age groups. However, COVID-19 differs in that presymptomatic and early asymptomatic transmission is well documented [15]. Consequently, preventive measures initiated only after symptom onset may be insufficient, leading to higher transmission rates among adults and older adults for COVID-19 compared with influenza.

In addition to these behavioral factors, differences in healthcare-seeking and testing behavior across age groups may have influenced the observed transmission patterns. Children often experience mild or asymptomatic infection and may be less likely to seek medical care, potentially resulting in under-ascertainment of secondary cases. Conversely, during outbreaks of acute respiratory infections in schools and preschools in Japan, children may undergo testing more frequently, which could lead to higher detection rates of influenza and COVID-19 in younger age groups. As a result, testing behavior is not uniform across ages and may bias estimates of household transmission in either direction.

With respect to infection prevention, the World Health Organization continues to emphasize the importance of fundamental public health measures, including physical distancing, mask use, adequate ventilation, and hand hygiene [16]. For influenza, the implementation of these measures within households has demonstrated effectiveness in limiting secondary spread. In contrast, the prevention of COVID-19 presents greater challenges given the well-documented occurrence of presymptomatic and early asymptomatic transmission. In such cases, traditional measures instituted after symptom onset are often insufficient. Prophylactic antiviral therapy has been proposed as a potential adjunct, as evidence suggests that early administration can suppress viral replication and reduce the likelihood of symptomatic progression [17,18]. Timely diagnosis also remains critical for mitigating transmission risk. The U.S. Centers for Disease Control and Prevention highlight that the high infectivity of SARS-CoV-2 during the presymptomatic phase necessitates rapid testing and immediate isolation at the earliest suspicion of infection or following close exposure [19,20]. Early case identification, combined with appropriate isolation, may substantially reduce secondary household cases.

Vaccination continues to represent the cornerstone of infection prevention for both influenza and COVID-19. By lowering susceptibility to infection and attenuating disease severity, vaccination indirectly reduces the probability of onward household transmission. The importance of vaccination is particularly pronounced for COVID-19 in older adults, among whom this study demonstrated elevated household transmission rates. Given their increased vulnerability to severe disease, vaccination provides dual benefits—limiting transmission potential while simultaneously reducing the risk of poor clinical outcomes in this high-risk population [21].

Exploratory analyses demonstrated that the interval to household transmission was generally shorter for influenza than for COVID-19. This finding aligns with established epidemiological evidence indicating that the serial interval of influenza—defined as the time between symptom onset in an index patient and a secondary case—is typically shorter than that of COVID-19 [13]. A systematic review of Omicron variants estimated the mean serial interval of COVID-19 at 3.2 days (95% confidence interval: 2.9–3.5 days) [13], whereas another review reported mean intervals of 2.2 days for seasonal influenza A(H3N2) and 2.8 days for pandemic influenza A(H1N1)pdm09 [14]. The concordance of the present findings with this established evidence supports the robustness and credibility of the transmission estimates derived from the current analysis.

An additional strength of this study lies in the application of real-world data to assess household transmission dynamics. The use of a large nationwide claims database enabled the evaluation of infection patterns as they occur under routine clinical conditions, providing a more representative picture of household-level transmission than might be achieved in controlled or limited study populations. The breadth of the dataset, encompassing diverse age groups and household structures, permitted detailed stratification by demographic. This comprehensive coverage facilitated the identification of nuanced patterns and risk factors that would likely remain obscured in smaller or more narrowly defined cohorts.

Although the claims database included basic demographic variables such as age and sex, it lacked many critical covariates necessary for adequate confounding control in household transmission analyses. Specifically, information on symptom severity, intensity and duration of household contact, infection control practices (e.g., mask use, isolation, hand hygiene, or ventilation), vaccination status, and the clinical indication for testing was not available. In addition, antiviral treatments could not be comprehensively ascertained, as oral antivirals were not reimbursed during part of the study period and monoclonal antibody therapies were not reimbursed and therefore not captured in claims data. The primary aim of this study was descriptive, and it was intended to characterize observed household transmission patterns within each disease rather than to estimate adjusted effects or to perform formal comparisons between diseases. Given the strong influence of age on household transmission, we presented age-stratified results to describe disease-specific patterns. These stratified analyses were intended to depict within-disease heterogeneity by age, rather than to serve as a basis for covariate adjustment or comparative modeling. Accordingly, multivariable adjusted analyses were not conducted, and the findings should be interpreted as descriptive patterns based on diagnosis sequence.

## 5. Limitations

This study has several limitations. First, household members were identified using insurance claim family codes, which do not provide definitive confirmation of cohabitation. Therefore, some cases classified as household transmission may have involved relatives that did not reside in the same household. Second, the determination of household transmission and identification of index patients were based on claim diagnoses, which may not accurately reflect all infections or the correct sequence of transmission. In this study, the index patient was defined as the first diagnosed individual within a household and does not necessarily represent the true primary case. Furthermore, the possibility of infections acquired outside the household cannot be excluded, limiting the certainty with which transmission events can be attributed solely to intrafamilial spread. Third, claims data do not include information on symptom severity or the clinical indication for testing. As a result, differences in healthcare-seeking and testing behavior across age groups could not be accounted for, which may have introduced differential detection bias in the estimation of secondary transmission rates. Fourth, the analysis did not incorporate information on infection control behaviors practiced by index patients or household members (e.g., mask use, isolation, or ventilation practices), nor did it account for antiviral therapy or vaccination status. Although age and sex were available in the dataset, several important determinants of infection and transmission risk—such as symptom severity, vaccination status, household contact intensity, and the indication for testing—were not captured in the claims data. Because the study was not designed to estimate adjusted effects or to formally compare transmission rates between diseases, multivariable regression analyses were not performed, and residual confounding cannot be excluded. The absence of these data constrains the interpretation of the observed transmission rates and represents a further limitation of the study. Most of the observation period analyzed in this study (November 2021 to August 2023) coincided with the implementation of Japan’s “Priority Preventive Measures” and multiple states of emergency, during which the public was strongly encouraged to refrain from nonessential outings. These circumstances may have created household contact environments that differ substantially from current conditions. Furthermore, influenza circulation was not observed in the 2021/22 season, and a smaller circulation was observed in the 2022/23 season [22] due to these interventions and other infection control measures targeting SARS-CoV-2. Given that this was an unusual period for respiratory virus transmission, the generalizability of the findings may be limited.

## 6. Conclusions

By quantifying household transmission rates of COVID-19 and influenza in relation to the age of index patients and their household cohabitants, this study provides a detailed assessment of intrafamilial spread based on diagnostic sequence. The findings underscore the importance of preventive measures targeting child-to-household transmission for both infections, as children were frequently identified as index patients in households with subsequent diagnoses, while also highlighting the need for interventions addressing transmission among adults and older adults in the context of COVID-19. Because SARS-CoV-2 can be transmitted prior to symptom onset, household preventive measures have inherent limitations. Therefore, early diagnosis, prompt treatment initiation, and—where feasible—prophylactic administration of antiviral agents is recommended. In addition, routine vaccination remains essential, both for reducing transmission potential and for mitigating the elevated risk of severe outcomes among vulnerable populations.

## Figures and Tables

**Table 1:** Baseline characteristics of index patients at Day 1.

Characteristic		COVID-19 *N* = 1,001,509 *n* (%)	Influenza *N* = 207,090 *n* (%)
Age (Years)	<12	273,901 (27.3)	120,269 (58.1)
	12–17	146,667 (14.6)	44,329 (21.4)
	18–29	136,119 (13.6)	20,119 (9.7)
	30–39	105,793 (10.6)	6922 (3.3)
	40–49	136,682 (13.6)	6957 (3.4)
	50–59	140,430 (14.0)	5819 (2.8)
	60–64	39,472 (3.9)	1740 (0.8)
	≥65	22,445 (2.2)	935 (0.5)
Number of household members	Mean	2.1	2.4
	SD	1.0	1.0
	Min	1	1
	Median	2.0	2.0
	Max	11	11
Sex	Male	600,601 (60.0)	121,907 (58.9)
Relationship	Insured	329,635 (32.9)	16,156 (7.8)
	Spouse of insured	121,979 (12.2)	7276 (3.5)
	Child	513,079 (51.2)	176,168 (85.1)
	Other/unknown	36,816 (3.7)	7490 (3.6)
Hospital type at diagnosis	Hospital	136,938 (13.7)	14,525 (7.0)
	General practice	864,453 (86.3)	192,533 (93.0)
	Unknown	118 (0.0)	32 (0.0)
Antiviral drug administration at Day 1	Yes	25,356 (2.5)	176,927 (85.4)
Time of diagnosis	1st quarter	139,425 (13.9)	31,453 (15.2)
	2nd quarter	394,821 (39.4)	10,904 (5.3)
	3rd quarter	223,193 (22.3)	11,130 (5.4)
	4th quarter	244,070 (24.4)	153,603 (74.2)

**Table 2:** Household transmission rates by age of index patients.

Age (Years)	COVID-19 *n*/*N* (%)	Influenza *n*/*N* (%)
<12	110,016/273,901 (40.17)	40,308/120,269 (33.51)
12–17	38,683/146,667 (26.37)	7183/44,329 (16.20)
18–29	24,395/136,119 (17.92)	1698/20,119 (8.44)
30–39	32,636/105,793 (30.85)	1447/6922 (20.90)
40–49	36,677/136,682 (26.83)	1196/6957 (17.19)
50–59	29,915/140,430 (21.30)	623/5819 (10.71)
60–64	8481/39,472 (21.49)	162/1740 (9.31)
≥65	5049/22,445 (22.49)	88/935 (9.41)

**Table 3:** Number of days to household transmission by age of index patients (days).

			COVID-19	Influenza
Total		*n*	285,852	52,705
		Mean (SD)	2.8 (1.6)	2.5 (1.4)
Age (Years)	<12	*n*	110,016	40,308
		Mean (SD)	2.8 (1.6)	2.4 (1.4)
	12–17	*n*	38,683	7183
		Mean (SD)	3.0 (1.6)	2.5 (1.4)
	18–29	*n*	24,395	1698
		Mean (SD)	2.9 (1.7)	2.5 (1.5)
	30–39	*n*	32,626	1447
		Mean (SD)	2.6 (1.6)	2.2 (1.4)
	40–49	*n*	36,677	1196
		Mean (SD)	2.7 (1.5)	2.2 (1.4)
	50–59	*n*	29,915	623
		Mean (SD)	2.7 (1.5)	2.4 (1.4)
	60–64	*n*	8481	162
		Mean (SD)	2.7 (1.5)	2.3 (1.4)
	≥65	*n*	5049	88
		Mean (SD)	2.7 (1.5)	2.6 (1.5)

SD—Standard deviation.

## Data Availability

De-identified data were obtained from the JMDC Claims Database under a data use agreement and are not publicly available. Analytic code is available upon reasonable request from the corresponding author.

## Supplementary Materials

The following supporting information can be downloaded at: https://www.mdpi.com/article/10.3390/v18030324/s1, Table S1: Household transmission rates by age of index patients and household members; Table S2: Interval from index diagnosis to household transmission by age of index patients and household members (days).

## Abbreviations

The following abbreviations are used in this manuscript:

COVID-19	coronavirus disease 2019
SARS-CoV-2	severe acute respiratory syndrome coronavirus 2
