# Supplementary material for: Household Transmission of COVID-19 and Influenza During the Early Omicron Era: An Insurance Claims Database Study in Japan
Source: Viruses. 2026 Mar 5;18(3):324. doi: 10.3390/v18030324 (PMC13030347; doi:10.3390/v18030324)
Supplement: Supplementary file 1 [file viruses-18-00324-s001.zip › viruses-4132613-supplementary.pdf]

Supplementary Tables

Supplementary Table S1. Household transmission rates by age of index patients and household members

COVID-19		**Age of household members**<br><br>**n / N (%)**							
		<12	12–17	18–29	30–39	40–49	50–59	60–64	≥65
**Age of index patients**	<12	57,494 / 187,164 (30.72)	11,984 / 57,606 (20.80)	6,477 / 24,792 (26.13)	48,331 / 172,973 (27.94)	41,913 / 177,258 (23.65)	4,965 / 23,914 (20.76)	172 / 1,013 (16.98)	232 / 2,401 (9.66)
	12–17	9,647 / 45,310 (21.29)	9,921 / 62,725 (15.82)	3,498 / 38,831 (9.01)	3,368 / 16,888 (19.94)	20,499 / 133,082 (15.40)	9,181 / 66,853 (13.73)	301 / 2,049 (14.69)	142 / 1,749 (8.12)
	18–29	6,838 / 28,915 (23.65)	3,481 / 36,657 (9.50)	5,215 / 45,540 (11.45)	1,126 / 4,518 (24.92)	4,991 / 49,664 (10.05)	9,802 / 107,541 (9.11)	889 / 9,140 (9.73)	169 / 2,002 (8.44)
	30–39	32,061 / 144,474 (22.19)	2,454 / 12,193 (20.13)	1,200 / 4,849 (24.75)	11,289 / 43,592 (25.90)	2,080 / 8,877 (23.43)	280 / 1,721 (16.27)	331 / 2,432 (13.61)	341 / 2,642 (12.91)
	40–49	20,490 / 101,857 (20.12)	13,037 / 81,568 (15.98)	3,255 / 30,090 (10.82)	2,472 / 10,618 (23.28)	11,822 / 60,335 (19.59)	2,398 / 12,823 (18.70)	90 / 548 (16.42)	533 / 4,740 (11.24)

	50–59	2,501 / 12,604 (19.84)	6,215 / 39,181 (15.86)	7,208 / 68,521 (10.52)	309 / 1,714 (18.03)	3,293 / 15,953 (20.64)	14,480 / 79,445 (18.23)	1,963 / 10,805 (18.17)	344 / 1,977 (17.40)
	60–64	64 / 429 (14.92)	210 / 1,107 (18.97)	811 / 6,458 (12.56)	285 / 2,070 (13.77)	98 / 510 (19.22)	2,774 / 12,405 (22.36)	3,807 / 18,511 (20.57)	972 / 4,929 (19.72)
	≥65	92 / 548 (16.79)	66 / 449 (14.70)	106 / 982 (10.79)	195 / 1,548 (12.60)	245 / 1,849 (13.25)	289 / 1,506 (19.19)	1,290 / 5,587 (23.09)	3,018 / 13,158 (22.94)

Influenza		**Age of household members**<br>**n / N (%)**							
		<12	12–17	18–29	30–39	40–49	50–59	60–64	≥65
**Age of index patients**	<12	29,043 / 94,404 (30.76)	5,266 / 25,880 (20.35)	1,540 / 9,382 (16.41)	12,106 / 76,885 (15.75)	9,368 / 77,932 (12.02)	1,021 / 9,739 (10.48)	42 / 428 (9.81)	41 / 981 (4.18)
	12–17	2,959 / 17,355 (17.05)	2,459 / 20,369 (12.07)	590 / 12,379 (4.77)	465 / 6,044 (7.69)	2,250 / 41,489 (5.42)	928 / 18,334 (5.06)	28 / 472 (5.93)	10 / 531 (1.88)
	18–29	512 / 3,194 (16.03)	466 / 6,968 (6.69)	328 / 7,207 (4.55)	35 / 471 (7.43)	302 / 8,796 (3.43)	524 / 16,751 (3.13)	39 / 1,183 (3.30)	5 / 250 (2.00)

	30–39	1,755 / 10,665 (16.46)	84 / 801 (10.49)	30 / 297 (10.10)	267 / 2,950 (9.05)	47 / 609 (7.72)	3 / 110 (2.73)	4 / 143 (2.80)	4 / 131 (3.05)
	40–49	932 / 6,414 (14.53)	396 / 3,985 (9.94)	75 / 1,449 (5.18)	44 / 556 (7.91)	227 / 3,102 (7.32)	30 / 615 (4.88)	2 / 30 (6.67)	8 / 195 (4.10)
	50–59	103 / 645 (15.97)	163 / 1,684 (9.68)	148 / 3,005 (4.93)	6 / 87 (6.90)	46 / 640 (7.19)	221 / 3,297 (6.70)	37 / 469 (7.89)	7 / 81 (8.64)
	60–64	7 / 26 (26.92)	6 / 56 (10.71)	11 / 269 (4.09)	11 / 95 (11.58)	4 / 21 (19.05)	40 / 466 (8.58)	72 / 827 (8.71)	22 / 287 (7.67)
	≥65	2 / 48 (4.17)	1 / 28 (3.57)	2 / 48 (4.17)	4 / 70 (5.71)	5 / 98 (5.10)	2 / 52 (3.85)	21 / 214 (9.81)	54 / 558 (9.68)

Supplementary Table S2. Interval from index diagnosis to household transmission by age of index patients and household members (days)

COVID-19			**Age of household members**							
			<12	12–17	18–29	30–39	40–49	50–59	60–64	≥65
**Age of index patients**	<12	n	57,494	11,984	6,477	48,331	41,913	4,965	172	232
		Mean (SD)	2.9 (1.7)	3.1 (1.7)	2.8 (1.6)	3.0 (1.7)	3.2 (1.7)	3.3 (1.7)	3.2 (1.7)	3.4 (1.7)
	12–17	n	9,647	9,921	3,498	3,368	20,499	9,181	301	142
		Mean (SD)	3.0 (1.7)	3.2 (1.7)	3.4 (1.8)	3.2 (1.7)	3.3 (1.7)	3.4 (1.7)	3.4 (1.8)	3.6 (1.9)
	18–29	n	6,838	3,481	5,215	1,126	4,991	9,802	889	169
		Mean (SD)	2.7 (1.6)	3.3 (1.8)	3.0 (1.7)	3.0 (1.6)	3.3 (1.7)	3.3 (1.7)	3.4 (1.8)	3.3 (1.6)
	30–39	n	32,061	2,454	1,200	11,289	2,080	280	331	341
		Mean (SD)	2.8 (1.7)	3.0 (1.7)	2.8 (1.5)	2.9 (1.6)	3.0 (1.6)	3.0 (1.6)	3.1 (1.8)	3.0 (1.6)
	40–49	n	20,490	13,037	3,255	2,472	11,822	2,398	90	533
		Mean (SD)	2.8 (1.6)	2.9 (1.7)	3.0 (1.7)	2.9 (1.6)	2.9 (1.6)	2.9 (1.6)	2.6 (1.4)	3.1 (1.7)
	50–59	n	2,501	6,215	7,208	309	3,293	14,480	1,963	344
		Mean (SD)	2.7 (1.6)	2.9 (1.6)	3.0 (1.7)	2.9 (1.6)	2.8 (1.5)	2.8 (1.5)	2.8 (1.5)	3.0 (1.5)
	60–64	n	64	210	811	285	98	2,774	3,807	972
		Mean (SD)	2.7 (1.8)	2.8 (1.7)	2.9 (1.6)	2.8 (1.5)	2.8 (1.6)	2.7 (1.4)	2.7 (1.4)	2.8 (1.6)
	≥65	n	92	66	106	195	245	289	1,290	3,018

		Mean (SD)	2.6 (1.6)	3.6 (1.9)	2.9 (1.7)	2.6 (1.5)	2.8 (1.7)	2.6 (1.4)	2.7 (1.4)	2.7 (1.5)

Influenza			**Age of household members**							
			<12	12–17	18–29	30–39	40–49	50–59	60–64	≥65
**Age of index patients**	<12	n	29,043	5,266	1,540	12,106	9,368	1,021	42	41
		Mean (SD)	2.6 (1.6)	2.6 (1.5)	2.8 (1.5)	2.9 (1.6)	2.9 (1.5)	2.8 (1.5)	3.1 (1.6)	3.0 (1.5)
	12–17	n	2,959	2,459	590	465	2,250	928	28	10
		Mean (SD)	2.6 (1.5)	2.6 (1.4)	2.7 (1.5)	3.0 (1.5)	2.9 (1.5)	2.9 (1.4)	2.8 (1.5)	2.9 (1.6)
	18–29	n	512	466	328	35	302	524	39	5
		Mean (SD)	2.5 (1.5)	2.6 (1.4)	2.6 (1.5)	2.7 (1.7)	2.8 (1.6)	2.9 (1.5)	3.0 (1.8)	2.6 (2.3)
	30–39	n	1,755	84	30	267	47	3	4	4
		Mean (SD)	2.5 (1.5)	2.5 (1.6)	2.5 (1.2)	2.7 (1.5)	2.9 (1.6)	3.0 (1.0)	3.0 (0.8)	3.8 (1.0)
	40–49	n	932	396	75	44	227	30	2	8
		Mean (SD)	2.4 (1.5)	2.5 (1.5)	2.5 (1.4)	2.6 (1.3)	2.8 (1.8)	2.6 (1.6)	2.0 (0.0)	3.3 (2.5)
	50–59	n	103	163	148	6	46	221	37	7
		Mean (SD)	2.6 (1.6)	2.5 (1.6)	2.4 (1.5)	2.5 (1.4)	2.1 (1.3)	2.5 (1.4)	2.4 (1.3)	2.1 (0.9)
	60–64	n	7	6	11	11	4	40	72	22
		Mean (SD)	1.7 (1.1)	1.8 (0.8)	1.7 (0.8)	2.4 (1.0)	3.0 (1.6)	2.4 (1.7)	2.3 (1.2)	2.7 (1.6)

	≥65	n	1	1	2	4	5	2	21	54
		Mean (SD)	3.0 (---)	1.0 (---)	2.5 (2.1)	1.5 (1.0)	3.0 (1.9)	2.5 (0.7)	2.5 (1.5)	2.7 (1.6)

SD, Standard deviation.

"---" indicates that SD could not be calculated due to insufficient sample size.
